# The sucrose challenge symptoms test optimized for diagnosis of congenital sucrase isomaltase deficiency

**DOI:** 10.1371/journal.pone.0310705

**Published:** 2024-09-18

**Authors:** Kasidy Street, Weng Tao, Brooks Cash, John Leung, Christopher Hayes, Derick Cooper, Ward Peterson

**Affiliations:** 1 QOL Medical LLC, Vero Beach, Florida, United States of America; 2 University of Texas Health Science Center, Houston, Texas, United States of America; 3 Boston Specialists, Boston, Massachusetts, United States of America; 4 Children’s National Hospital, Washington, DC, United States of America; University of Illinois at Chicago, UNITED STATES OF AMERICA

## Abstract

**Background:**

Congenital sucrase isomaltase deficiency (CSID), an inherited carbohydrate malabsorption disorder, is difficult to diagnose because of overlapping symptoms with other gastrointestinal (GI) diseases. An at-home study was conducted in CSID and healthy adults to evaluate the diagnostic utility of self-reported GI symptoms following administration of a sucrose challenge.

**Methods:**

This study investigated the optimum symptom scoring with a sucrose challenge symptoms test (SCST) for diagnosing CSID in 45 confirmed patients and 118 healthy controls. Subjects self-reported the severity of GI symptoms using a 10-point Likert scale after ingesting 50 grams of sucrose on an empty stomach. The receiver operator characteristics curve (ROC) was used to identify the diagnostic variable with the highest Youden Index, a measure of diagnostic performance.

**Results:**

All six symptoms were significantly worse in the CSID group within 2 hours after the sucrose challenge. The diagnostic variable with the highest Youden Index was worsening in global symptoms scores at 1- and 2-hours (11.7 [CSID] vs 3.2 [Controls]; *P*<0.001.) Optimized by gender, the sensitivity and specificity for this diagnostic variable were 87% and 81%, respectively.

**Conclusions:**

The SCST is a simple, non-invasive at-home test that can aid in a CSID diagnosis.

## Introduction

Sucrase-isomaltase (SI) breaks down sucrose and other disaccharides into monosaccharides that the body can absorb. Congenital sucrase-isomaltase deficiency (CSID) is a genetic disorder characterized by insufficient SI activity, resulting in post-prandial gastrointestinal bloating, pain, diarrhea, gas, borborygmi, and nausea [1]. Diagnosing CSID is challenging. Presenting symptoms overlap with irritable bowel syndrome, dyspepsia, and other carbohydrate intolerances and GI disorders [2, 3].

The historical gold standard for diagnosing CSID has been the disaccharidase assay, which measures activity in biopsies from an esophagogastroduodenoscopy [4, 5]. However, this assay is invasive, expensive, and not broadly accessible. In addition, it is subject to biopsy site variability, sampling and specimen handling errors and misinterpretation [6]. Non-invasive alternatives such as breath and genetic tests are fraught with other issues [3, 7, 8]. The sucrose challenge symptoms test (SCST) is based on the hypothesis that elicited GI symptoms following ingestion of a large dose of sucrose on an empty stomach can be used to diagnose CSID.

The present study was conducted to determine symptom scores, diagnostic variables, and cutoffs that can optimally distinguish CSID from healthy controls.

## Methods

### Participants and study design

This at-home study evaluated changes in self-reported symptoms severities following a sucrose challenge in confirmed CSID (experimental) and healthy (control) groups. The control group comprised individuals with no significant health problems or chronic GI symptoms. The experimental group was diagnosed with CSID (ICD-10 code E74.31) and treated with Sucraid^®^ (sacrosidase) Oral Solution 8500 IU/mL for at least one year at the time of informed consent. Prospective participants with non-CSID-related symptoms (e.g., small intestinal bacterial overgrowth, inflammatory bowel disease, celiac disease, etc.), recent medical procedures, and antibiotic use were excluded. The study was conducted by a single site with telemedicine capabilities and software to collect real-time data via mobile app. Participants were instructed to fast overnight before ingesting 50g sucrose dissolved in water. GI symptom severity was self-reported on a 10-point scale from 0 (no symptoms) to 9 (worst symptom severity) at baseline and every 30 minutes for 4 hours. The dose of 50g of sucrose was selected based on prior studies and clinical guidelines for carbohydrate malabsorption tests [2].

The study aimed to establish optimal cutoffs and reference ranges for symptom scores distinguishing CSID cases from controls. Symptom average and peak severity, total count, change-from-baseline, and time points were analyzed by group and gender. Receiver operator characteristics (ROC) curves assessed diagnostic test predictability using area-under-the-curve (AUC) and Youden Index to determine optimal sensitivity and specificity [9, 10]. This approach focused on effect estimation rather than hypothesis testing. A planned sample size of 50 CSID cases and 120 controls achieved 80% power to detect ≥1.5 differences in symptom severity means using a two-sided t-test with SD = 3.0.

### Ethics statement

This study was approved on July 22, 2021 by Advarra Institutional Review Board (approval number Pro00055707) with an approval expiration date of July 22, 2022. Recruitment occurred between August 30, 2021 and January 27, 2022. The study site scheduled and conducted a telemedicine appointment with each prospective participant to discuss the study. After consideration, willing participants provided electronic consent using a 21 CFR Part 11-compliant application by executing a handwritten signature using a computer mouse, touch screen, or stylus. The study site provided a countersignature in the same manner in real time prior to any study-related assessments. The informed consent process was documented by the study team and maintained as part of the participant’s source documentation in the data platform. Minors were not enrolled into this study.

## Results

**Fig 1:**
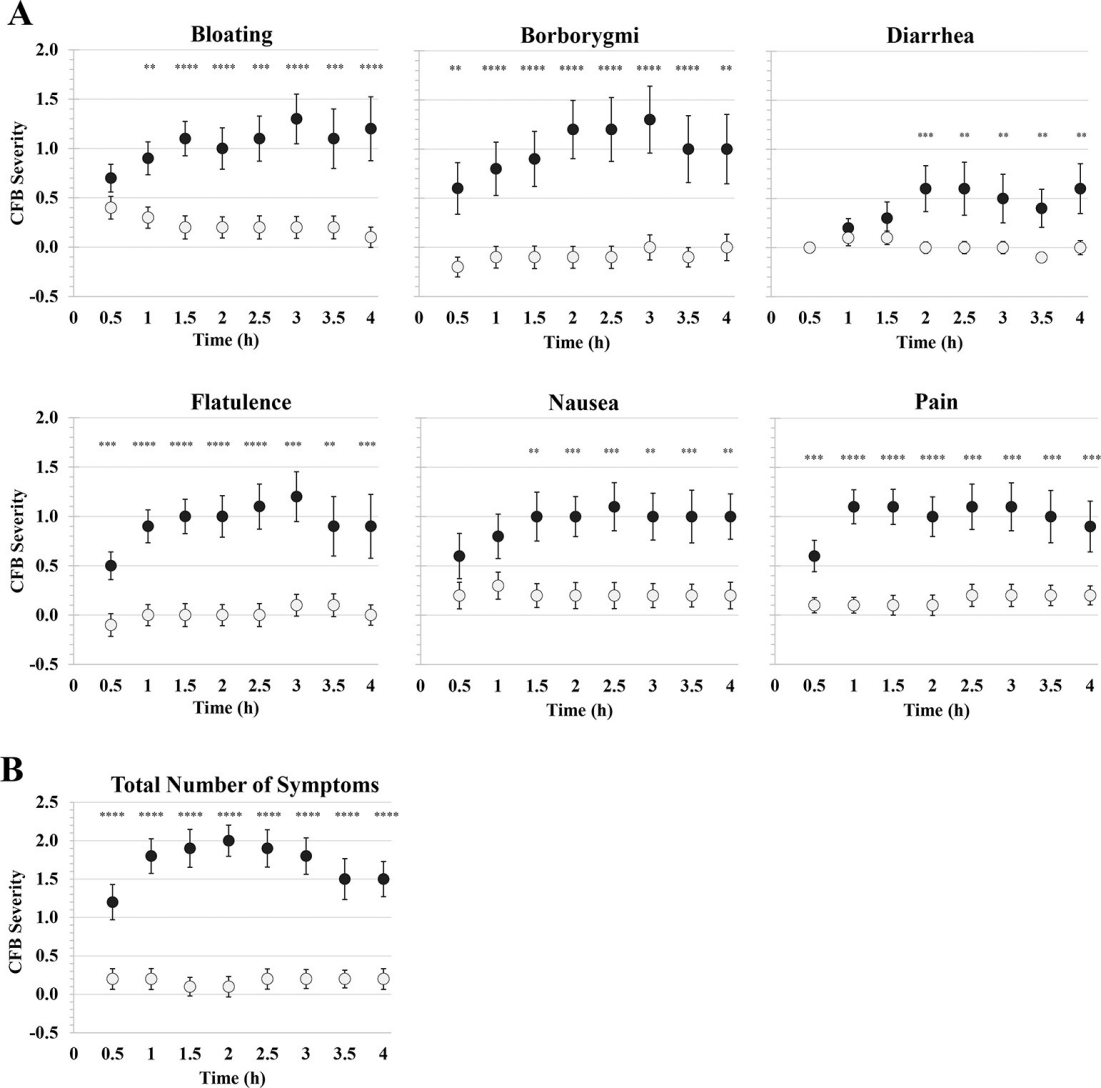
(A) Averaged self-reported change-from-baseline (CFB) severity scores for six GI symptoms by CSID group (black circles; N = 45) and control group (open circles; N = 118) at 0.5 to 4 hr following sucrose ingestion in 0.5-hr increments. (B) The total number of elicited symptoms in CSID and control groups. A symptom is defined as an “elicited symptom” if the self-reported symptom severity score is increased by at least 1 unit relative to baseline. Asterisks show p-values based on statistical comparisons between CSID and controls at each time point: *(p<0.05), **(p<0.01), ***(p<0.001), **** (p<0.0001).

Characteristics of CSID (n = 45) and control (n = 118) study participants are summarized in Table 1. Prior to sucrose ingestion, CSID cases were more symptomatic than controls, with significantly higher mean symptom severity scores for abdominal bloating (1.3 vs. 0.4, p<0.001) and flatulence/abdominal gas (1.1 vs. 0.5, p = 0.013), and a higher mean total number of symptoms (1.7 vs. 1.0, p = 0.017). Following sucrose ingestion, CSID patients reported significantly more symptoms (mean 4.5 vs. 2.3, p<0.001), a higher mean peak symptom severity overall (4.7 vs. 2.4, p<0.001), and a higher mean peak symptom severity for each of the individual symptoms. The symptom with the highest peak severity score in CSID cases was abdominal bloating (3.4 vs. 1.1, p<0.001). From 2 to 4 hours, the severity of all six symptoms and the total count of symptoms experienced were significantly greater in the CSID group (Fig 1).

**Table 1:** Characteristics of study participant by gender, race, age, weight, and height.

		Gender (%)	Race (%)				Age year (SD)			Weight lbs (SD)			Height in (SD)		
		Total	White	Asian	Black	Other	Avg	Min	Max	Avg	Min	Max	Avg	Min	Max
CSID	Female	38 (84)	35 (92)	1 (3)	0	2 (5)	47 (14)	20	80	158 (41)	107	293	65 (3)	60	70
	Male	7 (16)	7 (100)	0	0	0	42 (17)	19	63	159 (32)	123	205	70 (3)	66	74
	Overall	45 (100)	42 (93)	1 (2)	0	2 (4)	46 (15)	19	80	158 (40)	107	293	66 (4)	60	74
Control	Female	89 (75)	67 (75)	6 (7)	12 (13)	4 (4)	46 (14)	20	74	173 (51)	90	375	65 (3)	58	76
	Male	30 (25)	24 (80)	2 (7)	3 (10)	1 (3)	46 (15)	23	70	196 (39)	125	265	69 (3)	61	74
	Overall	119 (100)	91 (76)	8 (7)	15 (13)	5 (4)	46 (14)	20	74	179 (49)	90	375	66 (4)	58	76
P-value	Overall	0.267	0.065				0.951			0.006			0.73		

The diagnostic variable with the highest overall specificity and sensitivity for differentiating CSID from controls was a worsening in global symptoms score (GSS) at 1 and 2 hours (11.7 ± 10.06 vs 3.2 ± 7.09; *p*< 0.001). Table 2 shows gender specific cut-offs of five for females and three for males, which resulted in Youden Index, sensitivity, and specificity values of 0.64, 84%, and 80% (females) and 0.83, 100% and 83% (males). When separately optimized by gender and used to identify positive/negative cases, the resulting sensitivity and specificity were 87% and 81%, respectively, for the pooled population.

**Table 2:** 

	AUC	Youden Index	Sensitivity	Specificity	Cutoff	CSID Mean (SD)	Control Mean (SD)	p-value
**Female**	0.83	0.64	0.84	0.80	5	12.2 (10.78)	3.8 (7.88)	<0.001
**Male**	0.93	0.83	1.00	0.83	3	9.3 (4.19)	1.5 (3.55)	<0.001
**Combined**	--	0.67	0.87	0.81	5/3	11.7 (10.06)	3.2 (7.09)	<0.001

Summarized results from ROC analysis of global worsening in symptom severity scores at 1- and 2-hr by gender and group.

## Discussion

This study used self-reported severity scores to determine whether symptomatic responses following a sucrose load can be used to optimize a scoring method for diagnosing CSID. CSID volunteers consistently reported higher mean severity scores for all six symptoms (abdominal bloating, borborygmi, diarrhea, flatulence, nausea and abdominal pain) at all eight time points (0.5, 1, 1.5, 2, 2.5, 3, 3.5 and 4 hr following sucrose ingestion), and this difference is statistically significant from 2 to 4 hours. The diagnostic variable that best differentiates CSID cases from controls is the gender-specific worsening in GSS at 1 and 2 hr combined.

CSID can present with symptoms similar to those of IBS. Previous studies have shown that sucrase-isomaltase deficiency can be found in a significant proportion of IBS patients [11, 12]. The SCT could potentially be utilized to differentiate CSID from IBS and other conditions with overlapping symptoms. Future studies should focus on comparing the efficacy of SCST in distinguishing these conditions.

The current study does have some limitations. While the sample size approximated *a priori* power estimations, it fell slightly below the recruitment goals established at the outset of the trial. CSID females outnumbered CSID males by 5:1. The higher number of female subjects in both groups reflects the demographics of the accessible patient population for this study. Future research should aim for a more balanced gender representation. The diagnosis of CSID in the experimental group was not standardized. Furthermore, the selection of confirmed CSID patients as the experimental case group may result in a “nocebo” effect, in which patients’ expectations of symptoms worsening could lead to a perceived increase in symptoms unrelated to the actual ingestion of sucrose. Future studies should consider incorporating a non-sucrose sugar control to mitigate this bias. Finally, treatment with sacrosidase prior to a second SCT would potentially have increased diagnostic certainty regarding sucrose as a causal factor for the generation of GI symptoms among those patients with a positive sucrose challenge test.

This study nevertheless provides evidence to support an optimized GSS after a sucrose challenge as a non-invasive diagnostic tool for identifying adults with CSID. Sensitivities and specificities exceeding 80% were achieved based on self-reported scoring of six GI symptoms on a 10-point scale at only three time points (baseline, 1 and 2 hr). The SCST is non-invasive and does not require an in-clinic visit. Unlike the hydrogen-methane breath test, it is not dependent on measuring byproducts of commensal bacterial fermentation [2, 7]. No unintended or harmful adverse events were observed with the SCST. In conclusion, the SCST could serve as a useful, safe and practical diagnostic tool to help identify CSID patients.

## Supporting information

S1 Table Sucrose challenge symptoms data. (XLSX)
